# Comparison between a handheld ultrasound device and a traditional ultrasound for performing transcranial sonography in patients with Parkinson's disease

**DOI:** 10.1002/brb3.2891

**Published:** 2023-01-27

**Authors:** Maria A. S. Paes, Denise H. Nicaretta, Regina M. P. Alvarenga, Ana L. Z. Rosso, Rodrigo T. Brisson, Rita C.L. Fernandes

**Affiliations:** ^1^ Departamento de Radiologia Hospital Universitário Gaffrée e Guinle Rio de Janeiro Brazil; ^2^ Departamento de Neurologia Hospital Universitário Gaffrée e Guinle Rio de Janeiro Brazil; ^3^ Faculdade de Medicina Hospital Universitário Clementino Fraga Filho Rio de Janeiro Brazil

**Keywords:** handheld, Parkinson's disease, transcranial sonography, ultrasound

## Abstract

**Objective:**

The aim of this study is to compare a portable ultrasound (US) device and a traditional US for performing transcranial ultrasonography (CCT) in patients with Parkinson's disease (PD).

**Methods:**

This is a cross‐sectional, observational, and analytical study. The study recruited a total of 129 individuals from two public hospitals in the city of Rio de Janeiro in a prospective and non‐randomized manner between September 2019 and July 2021 as follows: group A with 31 patients with PD, group B with 65 patients with PD, and group C with 64 healthy individuals. Group A was used to collect data to establish the agreement analysis of the TCS measurements between the two devices. Groups B and C provided data for constructing the receiver operating characteristic curve for the handheld US. The subjects underwent the assessment of the transtemporal bone window (TW) quality, the mesencephalon area, the size of the third ventricle, and the substantia nigra (SN) hyperechogenicity area.

**Results:**

There was a good agreement between the methods regarding the quality of the TW–Kappa concordance coefficient of 100% for the right TW and 83% for the left, the midbrain area—intraclass correlation coefficient (ICC) of 69%, the SN area ICC = 90% for the right SN and 93% for the left and the size of the third ventricle ICC = 96%. The cutoff point for the SN echogenic area in the handheld US was 0.20 cm^2^.

**Conclusions:**

The handheld US is a viable imaging method for performing TCS because it shows good agreement with the measurements performed with traditional equipment, and the measurement of SN echogenic area for PD diagnosis presents good sensitivity and specificity.

## Graphical abstract

Graph 1. The receiver operating characteristic (ROC) curve. The area under the curve is 0.91 with a confidence interval (CI) of 95%: 0.84–0.97. The best substantia nigra cutoff value was 0.20 cm^2^. Sensitivity = 0.98 and specificity = 0.87.

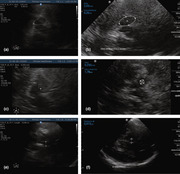

## 1 INTRODUCTION

Transcranial sonography (TCS) is a neuroimaging technique used to obtain and evaluate images of the cerebral parenchyma in movement disorders through B‐mode and assess the blood flow velocities of the intracranial vessels through Doppler mode (Brisson et al., 2021; Monaco et al., 2018). This is an ultrasonographic technique used to detect abnormalities in the echogenicity of substantia nigra (SN), thalamus, lenticular nuclei, red nuclei, the continuation of the median raphe nucleus, and ventricular diameters and assesses vascular reactivity (Brisson et al., 2021; Monaco et al., 2018; Walter et al., 2008). The main limitation of TCS is the lack of bone window, which could vary between 5% and 44% of the population, according to Brisson, Santos et al. (2021), depending on various factors, including, sex, age, and ethnicity.

The B‐mode on TCS as a tool to assist in the diagnosis of Parkinson's disease (PD) appeared in 1995 when Becker et al. (1995) noticed the increase in echogenicity of the SN for the first time in a group of patients with PD. Since then, the method has been the object of study of innumerable research studies, primarily in Europe, the Americas, Japan, and Korea, with the first publication in Brazil by Fernandes et al. (2011). Currently, the use of TCS as an auxiliary method in PD diagnosis using the B‐mode scan is already well established.

TCS has numerous advantages over other neuroimaging methods. As it is non‐invasive, it does not emit ionizing radiation; it is performed in real‐time; it has a short duration and relatively low cost, with no loss due to movement; and, above all, can be performed at the bedside as many and necessary times as possible (Ali et al., 2018; Berg et al., 2008).

PD, the main cause of parkinsonism, is a slow, asymmetrical, idiopathic progressing disease, resulting in most of the progressive loss of neurons in the ventrolateral portion of the pars compacta of the SN. PD is characterized by the presence of bradykinesia and at least one of the two cardinal signs: rigidity and resting tremor, usually occurring in persons that are 50 years old or older. The diagnosis continues to be challenging when the motor signs appear, and at this point, complementary exams can help (Hughes et al., 2002; Postuma et al., 2015).

SN hyperechogenicity is observed in about 90% of PD, with cutoff values between 0.20 and 0.25 cm^2^ in most studies depending on the ultrasound (US) system used (Berg et al., 1999, 2001). This image correlates with cellular iron and neuromelanin deposition and the microglia activation mechanism demonstrated in postmortem studies (Berg et al., 2010; Zecca et al., 2005).

Almeida et al. (2022) in their research observed an estimated accuracy of 79.2% for the PD diagnosis by the TCS, while the 99mTc‐TRODAT‐1 DATSPECT was associated with an accuracy of 99%. This method has a significantly higher diagnostic accuracy for PD than TCS. However, sonography does not use radiation ionizing or radiopharmaceuticals, has a lower cost, and is more available in health services, which makes this method an interesting tool.

With the advent of technological advances in recent decades, the US has become increasingly portable. More recently, new pocket US devices have become available, making it possible to use the handheld US, which brings ease of transport of the device to perform the exam (Ali et al., 2018). The handheld US has advantages over traditional equipment, the main one being portability as it is much smaller and lighter, which allows it to be easily transported to hard‐to‐reach places; another difference is the lower cost of its acquisition.

This research aimed to verify the agreement between a handheld US device and a traditional US for performing TCS in patients with PD. Another research objective was to establish the SN hyperechogenicity area cutoff point for the handheld US. Considering this information, we did not find studies in the literature that evaluated the use of handheld US for performing TCS in patients with PD.

## 2 METHODS

### 2.1 Study population

This is a cross‐sectional, observational, and analytical study where 129 individuals were recruited, non‐randomized, between September 2019 and July 2021 and divided into three groups. Group A with 31 PD patients from the Movement Disorders Outpatient Clinic of the Clementino Fraga Filho University Hospital of the Federal University of Rio de Janeiro (MDOC‐CFFUH‐FURJ), group B with 65 PD patients from MDOC‐HUCFF‐FURJ and the PD outpatient clinic of the Gaffrée and Guinle University Hospital of the Federal University of the State of Rio de Janeiro/ Empresa Brasileira de Serviços Hospitalares (CPD‐GGUH‐FUSRJ/EBSERH), and group C with 64 healthy individuals, recruited among GGUH‐FUSRJ /EBSERH workers.

Clinical and sociodemographic data were collected using a semi‐structured questionnaire.

The collected data from group A was used to establish the agreement analysis of the TCS measurements between the two devices: Philips Lumify S4‐1 (Koninklijke Philips B.V.) handheld US, which has an app‐based US, 4–1 MHz, phased array transducer that was used in association with Samsung Galaxy Tab S4 10.5″ and traditional equipment Philips HD11.XE/HD11 (Philips Medical Systems), equipped with an S4‐2, 4–2 MHz, phased array transducer. TCS was performed in this group, first by examiner 1 with the traditional equipment and after by examiner 2 with the handheld US, and one did not have access to the other's test results and vice versa. These data were statistically analyzed by the intraclass correlation coefficient (ICC) to verify the agreement of the measurements performed in the two systems.

Groups B and C provided data for the construction of the receiver operating characteristic (ROC) curve for the handheld US.

PD patients inclusion criteria in groups A and B: The PD patient's inclusion criteria are age over 18 years, with a minimum of 2 years of PD diagnosis and 5 years of follow‐up. The diagnosis was performed following the MDS‐PD 2015 criteria confirmed by two neurologists with more than 5 years of experience in movement disorder diseases.

PD patients exclusion criteria in groups A and B: Presence of atypical and secondary parkinsonism and previous neurosurgery.

Inclusion and exclusion criteria in group C: Age over 18 years of age and absence of neurodegenerative diseases, hyposmia or anosmia, previous stroke, and neurosurgery.

### 2.2 US examination techniques and TCS protocol

The US technique consists of placing the probe on the temporal region in front of the auricle of the ear, on the orbitomeatal line of the hemifacial region, and then searching for the temporal bone acoustic window, the thinnest region of the bone plate that allows the passage of low‐frequency ultrasonic pulse.

The subjects were placed in a dorsal decubitus position and the examiner, on the right, in a supine position. To acquire images of structures of interest in the midline area bilaterally, the examiner then made circular movements with the probe over the TW.

The parameters for adjustment were the same on both equipment: frequency of 2 to 4 MHz, a dynamic range of 45 to 55 dB, and a penetration depth of the ultrasonic beam between 14 and 16 cm, contour amplification medium or high. Image brightness and time gain compensation were adjusted according to need. Both US systems had a phased array transducer (Koloudk et al., 2009; Walter & Skoloudik, 2014; Walter et al., 2007).

The subjects were evaluated for the quality of the TW, the mesencephalon area, the size of the third ventricle, and the SN hyperechogenicity area.

The criterion used for the insufficient window was the non‐visualization of the SN and midbrain. The insufficient bilateral window was an exclusion criterion for SN and midbrain analysis. However, in all cases, we evaluated the window quality parameter, and in some cases of bilateral insufficient window (for SN and midbrain), it was possible to visualize the third ventricle.

The measurements acquired were: the midbrain area in square centimeters on the axial plane, which, in the handheld US, was calculated using an ellipsis that allowed manual adjustment, which is the only method available to calculate area on this machine; and in the traditional US, it was manually circumscribed, the method available to calculate area on this equipment. The hemi‐midbrain area ipsilateral was measured on both sets of equipment and multiplied by two to obtain the value of the total area of the midbrain.

Calculation of the SN hyperechogenicity was performed the same way as the measurement of the midbrain area on both sets of equipment. Hyperechogenicity of SN is defined by increased size, compared with normal ranges, and the basal cisterns were the referential used to identify the SN hyperechogenicity.

The largest transverse diameters of the third ventricle were measured. The size of the third ventricle was calculated in centimeters by measuring the distance between the linear hyperechoic inner layers of the ependyma, on the axial plane, ipsilateral, at the level of the thalamus (Walter et al., 2007). This technique was identical on both machines.

**FIGURE 1:**
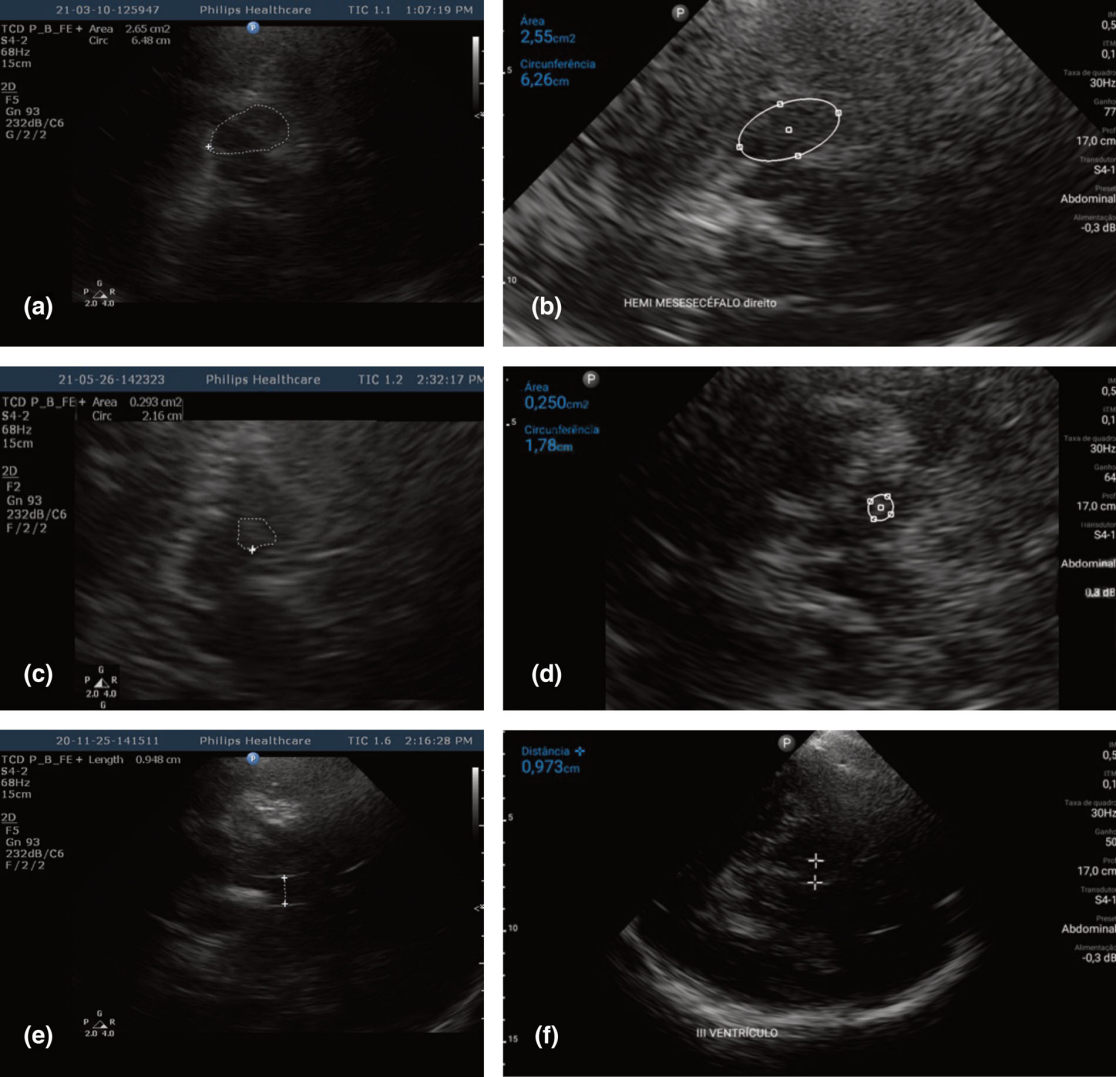
Axial transcranial ultrasound images of the same patient with Parkinson's disease show similar values of measurements acquired by the two US systems. Left row images (A‐C‐E) acquired with traditional equipment. The images of the right line (B‐D‐F) were acquired with the handheld US. (A) TCS in traditional equipment shows the manual delimitation of the right hemisencephalon to calculate the area. (B) TCS on handheld US shows that the right hemisencephalon area was calculated by an ellipse. (A and B) ‐ Measurements of the hemisencephalon area: 2.65 cm^2^ and 2.55 cm^2^. (C) TCS in traditional equipment shows that manual delimitation circumscribes the echogenic area of the left SN. (D) TCS on handheld US shows that the echogenic area of the left SN was calculated by an ellipse. (C and D) – The echogenic area of the ipsilateral substantia nigra (SN): 0.293 cm^2^ and 0.250 cm^2^. (E) TCS in traditional equipment shows the measurement of the third ventricle. (F) Handheld US TCS shows the measurement of the third ventricle. (E and F) Diameter of the 3rd transverse ventricle: 0.948 cm and 0.973 cm.

All the measurements were acquired after freezing and expansion of the images (Figure 1).

The reference value for the third ventricle according to Huber (2010) for individuals under 60 years of age is less than 0.7 cm and for individuals more than 60 years is less than 1.0 cm.The reference value for the entire midbrain area according to Aoun et al. (2021), by an experienced TCS rater, can have a mean value of 4.47 ± 0.53 cm^2^. The cutoff values of SN hyperechogenicity for PD can vary between 0.20 and 0.25 cm^2^ depending on the US system used (Berg et al., 1999, 2001).

The exams were performed by two sonographers (with more than 5 years of experience with the method and more than 2 years with TCS). In group A (sample of 31 patients with PD), each individual was evaluated at the same time by examiner 1 and then by examiner 2. Examiner 1 used traditional equipment, and examiner 2 used the handheld US. One examiner did not have access to the other examiner's images or measurements. Groups B (all PD patients) and C (healthy individuals) were evaluated only by examiner 2 with the handheld US.

### 2.3 Statistical analysis

Data gathered have been analyzed using the R Project for Statistical Computing version 4.1.3 and Statistical Analysis Software version 9.4. An exploratory analysis of data was carried out using measurements of central tendency and dispersion. Taking into consideration the absolute and relative frequencies, the qualitative variables were expressed in mean ± standard deviation, and the categorical variables were expressed as a percentage. For comparisons between the groups, we used chi‐square and Mann–Whitney tests. The accepted level of statistical significance was established at 5%.

The verification of concordance between the measurements performed in the handheld US and traditional equipment was calculated using the Kappa concordance coefficient (KCC) for categorical variables and the ICC for qualitative variables.

To verify the homogeneity between the distribution of the data from the groups—PD cases and healthy control subjects—regarding the clinical variables, a chi‐square test and a non‐parametric Mann–Whitney test were performed.

The ROC curve and the Youden index were calculated to identify the cutoff for the SN area trough on the handheld US. The SN hyperechogenicity area cutoff point for differentiating PD from healthy individuals was established according to the 90th percentile of the hyperechoic area in the SN of the healthy group (group C). The SN's largest unilateral area was used for the construction of the ROC curve.

## 3 RESULTS

Among the 129 participants recruited, 65 presented with PD, and 64 were healthy control subjects. There was a predominance of males (57.36%) and the median age was 68 [58, 74] years old for parkinsonians and 58 years old [51.5, 62.5] for the control group, with *p* < .001. The mean duration of PD was 8 [6, 10] years. (Tables 1 and 2).

**TABLE 1:** Distribution of qualitative variables pertaining to the total population of the study

Variables		
** Hospital**	No.	%
GGUH‐FUSRJ	98	75.97
CFFUH‐FURJ	31	24.03
**Gender**	No.	%
Female	55	42.64
Male	74	57.36
**Color**	No.	%
White	71	55.04
Not White	58	44.96
**Diagnosis**	No.	%
Parkinson's disease (PD)	65	50.39
Healthy individuals	64	49.61

**TABLE 2:** Distribution of the variable—Age—in PD patients and healthy individuals (without PD)

Variable	Diagnosis	No.	Mean	Standard deviation	Median	Q1	Q3	Minimum	Maximum
Age	With/ and without/PD	129	62.47	1.92	62	53	72	19	91
Age	PD	65	66.51	12.27	68	58	74	19	91
Age	Without PD	64	58.36	10.09	58	51.5	62.5	39	91

### 3.1 TCS with the handheld US for the ROC curve

The area under the ROC curve was 0.91 with a confidence interval of 0.95 (lower limit 0.84 andupper limit 0.97) The Youden index was 0.85 with the measure of 0.20 cm^2^ representing the best cutoff pointfor the SN hyperechogenicity area to differentiate between sick (with PD) and healthy individuals in this sample (Table 3). The sensitivity of the test was 98.3% and specificity 86.7%. The positive predictive value (PPV) was 0.89, and the negative predictive value (NPV) was 0.9898%.

**TABLE 3:** Best cutoff points of the substantia nigra (SN) hyperechogenicity area according to the receiver operating characteristic curve

	Best cutoff point	Sensitivity	Specificity	PPV	NPV
Right SN (RSN)	0.19	0.983	0.630	0.744	0.971
Left SN (LSN)	0.2	0.983	0.630	0.740	0.971
Dominant SN	0.2	0.980	0.870	0.890	0.98

### 3.2 TCS with handheld US—Evaluation of TW

The handheld US evaluated all participants’ quality of the transtemporal bone window. Sixteen participants (12.4%) had insufficient TW (11 PD patients, 16.92%, and five of the healthy control group, 7.81%; Table 4).

**TABLE 4:** Distribution of the transtemporal bone windows (TWs) in all individuals examined—PD patients and healthy individuals (without PD)—by transcranial sonography

TW	PD	Without PD	Total
Absent	11	5	16
	16.92%	7.81%	(12.4%)
Unilateral	0	1	1
	0	1.56%	(0.78%)
Bilateral	54	58	112
	83.08%	90.63%	(86.82)
Total	65	64	129

Group A was examined by the TCS with both handheld and traditional systems. All of them had the window quality parameter considered. Only 28 patients participated in the analysis of agreement between the SN and midbrain measurements because three had insufficient bone windows. However, all of them allowed the analysis of the third ventricle.

In groups B and C, all patients had the quality parameter of the windows evaluated. In group B, 11 patients had absent windows for SN and midbrain, and only 61 patients were possible to evaluate the third ventricle. In group C, five of them had no window for SN and midbrain but was possible to evaluate the third ventricle in 61 individuals.

No subject was excluded from the study because all of them had at least the quality of the bone window evaluated.

The right SN (RSN) was assessed in the 113 subjects with sufficient TW, there being 59 of the healthy control group. In the PD patients, the mean value of the RSN was 0.18 cm^2^ and 0.04 cm^2^ in the control group, *p* < .0001. The left SN (LSN) was assessed in 112 subjects with permissive TW, there being 58 from the control group. In the PD group, the mean value of the LSN was 0.19 cm^2^ and 0.04 cm^2^ in the healthy control group, *p* < .0001 (Table 5).

**TABLE 5:** Association between PD patients and healthy individuals (without PD)—with the quantitative variables

Variable	Diagnosis	No.	Mean	Standard deviation	Median	Q1	Q3	Minimum	Maximum	*p*‐value*
Midbrain area	PD	54	4.78	0.51	4.6	4.4	5.2	4	5.90	.3933
	Without PD	59	4.84	0.48	4.8	4.5	5.1	4	6.20	
Third ventricle	PD	61	0.55	0.22	0.6	0.4	0.70	0.10	1.10	.0041
	Without PD	61	0.44	0.16	0.4	0.3	0.50	0.20	0.90	
RSN	PD	54	0.18	0.13	0.24	0	0.27	0	0.39	<.0001
	Without PD	59	0.04	0.07	0	0	0.11	0	0.34	
LSN	PD	54	0.19	0.14	0.25	0	0.28	0	0.44	<.0001
	Without PD	58	0.04	0.09	0	0	0.09	0	0.45	

* *p*‐value pertaining to the Mann–Whitney test.

### 3.3 TCS for analysis of agreement between systems

The assessment of the right TW performed with the handheld US agreed 100% with traditional equipment. Of the 31 subjects from the A group examined with both equipment, it was observed that three subjects presented with insufficient right windows. The analysis of the inter‐system agreement for the left TW reached a KCC of 83%. Handheld US categorized 27 subjects with sufficient left TW, while traditional equipment described 28 subjects as sufficient (Tables 6 and 7).

**TABLE 6:** Transcranial sonography intersystem to assess the quality of TW performed by examiners 1 and 2

Variable	No.	%
Right TW 1		
Insufficient	3	9.68
Sufficient	28	90.32
Left TW 1		
Insufficient	4	12.90
Sufficient	27	87.10
Right TW 2		
Insufficient	3	9.68
Sufficient	28	90.32
Left TW 2		
Insufficient	3	9.68
Sufficient	28	90.32

*Note*: Distribution of concordance sample windows between systems in subjects with PD.

**TABLE 7:** Transcranial sonography intersystem to assess the quality of TW performed by examiners 1 and 2

	Right TW 2			CI 95%	
Right TW 1	0	1	KCC	LL	UL
0	3 (9.68)	0 (0)	1	1	1
1	0 (0)	28 (90.32)			

	Left TW 2			CI 95%	
Left TW 1	0	1	KCC	LL	UL
0	3 (9.68)	1 (3.23)	0.839	0.534	1.000
1	0 (0)	27 (87.1)			

*Note*: Kappa concordance coefficient (KCC) where lower limit (LL) and upper limit (UL) with confidence interval (CI) for windows are shown in subjects with PD.

The midbrain area was measured in 28 subjects from the PD group with sufficient TW. In the handheld US, the mean value of the measurement of the mesencephalon area was 4.8 cm^2^, while in traditional equipment, it was 4.7 cm^2^. The inter‐system agreement was 69%, calculated by the ICC (Tables 8 and 9).

**TABLE 8:** Distribution of the quantitative variables in the group for intersystem assessment

Variable	No.	Mean	Standard deviation	Median	Q1	Q3	Minimum	Maximum
Midbrain area 1	28	4.8	0.5	4.6	4.4	5.2	4.0	5.9
Third ventricle 1	31	0.6	0.2	0.6	0.3	0.7	0.1	1.0
RSN 1	28	0.2	0.1	0.2	0.0	0.3	0.0	0.4
LSN 1	28	0.2	0.1	0.3	0.0	0.3	0.0	0.4
Midbrain area 2	28	4.7	0.5	4.6	4.4	5.0	4.0	6.0
Third ventricle 2	31	0.6	0.2	0.6	0.3	0.7	0.2	1.0
RSN 2	28	0.2	0.1	0.2	0.0	0.3	0.0	0.4
LSN 2	28	0.2	0.1	0.2	0.1	0.3	0.0	0.4

**TABLE 9:** Intraclass correlation coefficient (ICC) where LL and UL with CI of the quantitative variables in the group for intersystem assessment

		CI 95%	
Variable	ICC	IL	UL
Midbrain area	0.69	0.44	0.83
Third ventricle	0.96	0.94	0.98
RSN	0.90	0.82	0.95
LSN	0.93	0.86	0.96

The third ventricle had its thickness checked in 31 subjects with permissible TW. In both systems, the values found were the mean and the median, 0.6 cm, and the ICC, 96% (Tables 8 and 9).

In 28 subjects from the PD group, where the RSN was assessed, both systems had mean and median values of 0.2 cm^2^ and an ICC of 90%. The LSN was verified in 28 subjects from the PD group, where the handheld US measured a mean of 0.2 cm^2^ and a median of 0.3 cm^2^, while the measurements of traditional equipment were mean and median of 0.2 cm^2^ and the ICC was 93% (Tables 8 and 9).

## 4 DISCUSSION

This study verifies the agreement between a handheld US device and the traditional US for performing TCS in patients with PD, and as far as we know, it is the first study that performs this analysis. For that, it was necessary to construct the ROC curve for the handheld US, which reveals high values of the sensitivity (98.3%), specificity (86.7%), Youden index of 0.85, and the SN hyperechogenicity area of 0.20 cm^2^ as a proper cutoff point. When we compared both devices, we identified a strong concordance value between the methods, mainly about TW quality (KCC of 100% for the right TW and 83% for the left), the size of the third ventricle (ICC = 96%), and the SN (ICC = 90% for the RSN and 93% for the LSN). The measurement of the midbrain area showed the least concordance value between the equipment (ICC = 69%). The percentage of TW insufficiency in the study sample was 12%.

The results infer that the handheld US is a valuable tool for assisting in the diagnosis of PD as the traditional equipment and therefore a positive response to the study, in keeping with our objective. Toscano et al. (2020) compared the same handheld US used in this study with other non‐portable equipment in the area of gynecology and corroborating our result concluded that the handheld US has good accuracy and has potential applicability in routine medical practice.

The lowest concordant value of inter‐systems measured was that of the midbrain area, it can perhaps be deduced that the midbrain, as it is a sinuous structure, makes it difficult to be measured using a system that is not 100% manual, like the software available for measuring in traditional equipment. The lack of such a manual program for measuring the area is probably a negative factor for this handheld US (Lumify). In addition, a possible update from the manufacturer to arrive at a manual measuring model, like the one in traditional equipment, could be interesting.

T he ROC curve and Youden index for the handheld US, revealing high values of the sensitivity, specificity, and the area of 0.20 cm^2^ as an SN hyperechogenicity area cutoff point to differentiate the individuals with PD from the control subjects using TCS. This significant result refers to one of the research objectives. The cutoff obtained was similar to other studies published with traditional machines from various manufacturers and in different countries, given that the majority of the studies worldwide show that an optimal cutoff is between 0.20 and 0.25 cm^2^. Vivó‐Orti et al. (2013) in their research used traditional equipment and found the area of 0.22 cm^2^ as a cutoff point in the 90th percentile, and the ROC curve was constructed with 75 subjects with PD and 91 healthy individuals. Xu et al. (2020), in their research, made two ROC curves, one for each traditional equipment used to discriminate subjects with PD (*n* = 278) from healthy individuals (*n* = 300) to measure SN hyperechogenicity. The cutoff points found were 0.20 and 0.21 cm^2^ for each respective piece of equipment. The current study with the handheld US found a very similar cutoff to these two studies performed with non‐portable equipment and agrees with the values found in most studies worldwide.

The main limitation of studies about TCS is the insufficiency of TW in part of the population, in which this research revealed the percentage in the studied population of 12.4%, a very similar result to another Brazilian study that found 10.8% of inadequate TW (Fernandes et al., 2011). These statistical data are extremely important to build population epidemiological data about the viability of TW, considering that there are still few original Brazilian research studies.

Another limitation is the size of the sample used to assess the inter‐system agreement, even so, the study obtained consistent statistical results. The study could not also use only one type of traditional equipment for agreement reference for the handheld US.

## 5 CONCLUSION

The handheld US is a viable imaging method for performing TCS because it shows good agreement with the measurements performed with traditional equipment and because its ROC curve and Youden index demonstrate adequate cutoff of the SN area in patients with PD. The handheld US can be a valuable, practical, fast, bedside method and have the potential to be a cost‐effective solution to assist in the diagnosis of PD.

## CONFLICT OF INTEREST

The authors declare that they have no conflict of interest.

### PEER REVIEW

The peer review history for this article is available at https://publons.com/publon/10.1002/brb3.2891

## Data Availability

The data that support the findings of this study are available from the corresponding author upon reasonable request.
